# Targeting Versican as a Potential Immunotherapeutic Strategy in the Treatment of Cancer

**DOI:** 10.3389/fonc.2021.712807

**Published:** 2021-08-30

**Authors:** Priyanka Hirani, Valentine Gauthier, Carys E. Allen, Thomas N. Wight, Oliver M. T. Pearce

**Affiliations:** ^1^Centre for Tumour Microenvironment, Barts Cancer Institute, Queen Mary University of London, London, United Kingdom; ^2^Matrix Biology Program, Benaroya Research Institute at Virginia Mason, Seattle, WA, United States

**Keywords:** tumour microenvironment, tumour immunity, immunotherapy, versican, matrisome

## Abstract

A growing body of literature links events associated with the progression and severity of immunity and inflammatory disease with the composition of the tissue extracellular matrix as defined by the matrisome. One protein in the matrisome that is common to many inflammatory diseases is the large proteoglycan versican, whose varied function is achieved through multiple isoforms and post-translational modifications of glycosaminoglycan structures. In cancer, increased levels of versican are associated with immune cell phenotype, disease prognosis and failure to respond to treatment. Whether these associations between versican expression and tumour immunity are the result of a direct role in the pathogenesis of tumours is not clear. In this review, we have focused on the role of versican in the immune response as it relates to tumour progression, with the aim of determining whether our current understanding of the immunobiology of versican warrants further study as a cancer immunotherapy target.

## Introduction

In recent years, immunotherapy of cancer has achieved impressive clinical benefits (1). By targeting the mechanisms underlying tumour immune evasion, cancer immunotherapy aims to stimulate and reactivate the ability of the immune system to detect and eradicate cancer cells. Established therapies use a range of manipulations to strengthen host anti-tumour immunity, such as cancer vaccines to induce antigen-specific immunisations; oncolytic viruses to enhance the immunogenicity of the tumour; administration of immunologic adjuvants, cytokines or immunomodulators to activate innate and adaptive immune and inflammatory pathways within the tumour microenvironment (TME); T cell transfer therapy such as Chimeric Antigen Receptor (CAR) T cell therapy to exclusively boost tumour-specific T cells and reactivate tumour-infiltrating lymphocytes; and finally monoclonal antibodies to specifically disrupt immune regulatory mechanisms hijacked by tumour cells (2). Notably, anti-CTLA4 and anti-PD-1/L1 immune checkpoint blockade (ICB) antibodies have demonstrated long-term remissions in patients with advanced staged tumours and are now emerging as frontline treatment for solid cancers including metastatic melanoma, non-small cell lung cancer, renal cell carcinoma and bladder urothelial cancer (3).

The success of immunotherapies remains limited to a subset of individuals (4), with most either displaying resistance or acquired resistance to these therapies (5). Therefore, elucidating the differences between responders and non-responders is key to patient selection, perhaps though the identification of biomarkers and the development of approaches to improve immunotherapy response rates.

**Figure 1:**
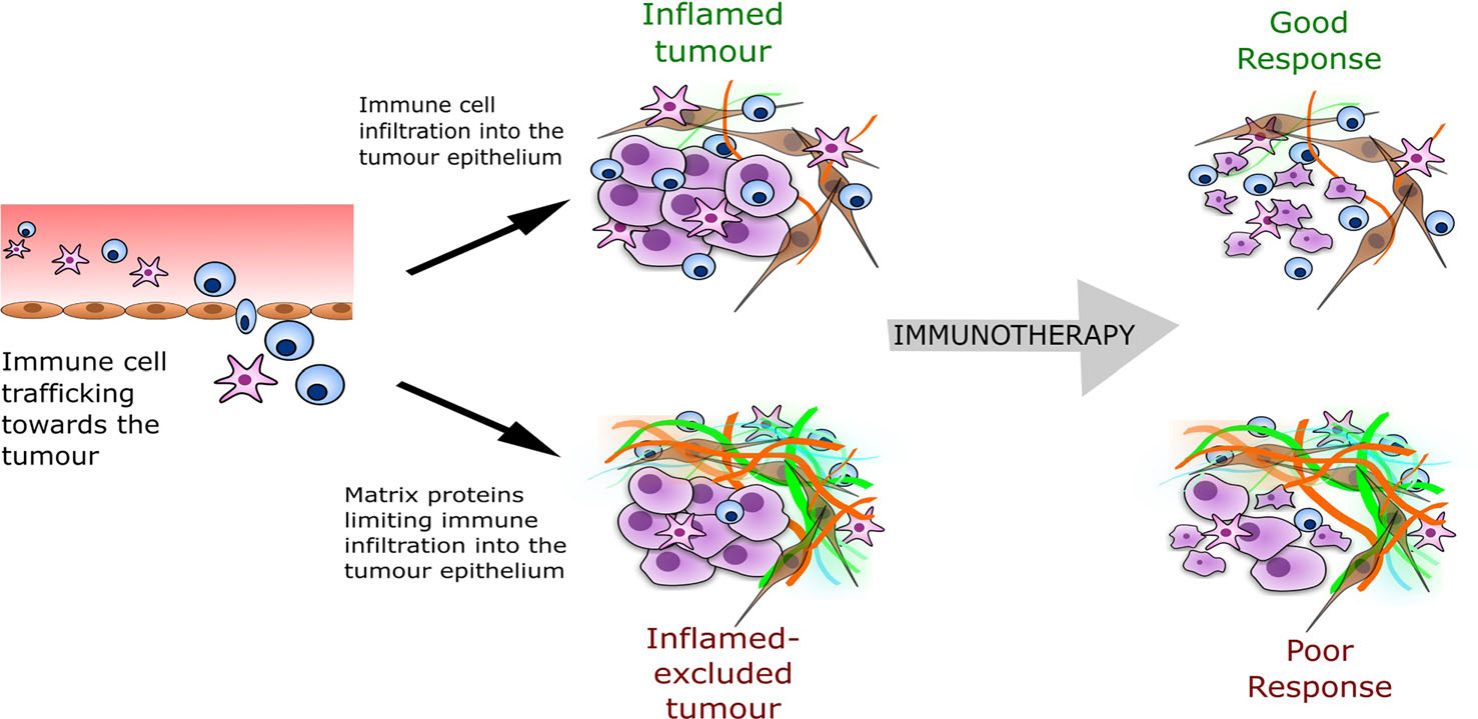
The tumour immune phenotype and predicted response to immunotherapy. The inflamed-excluded phenotype is defined through less interaction or contact of cytotoxic immune cells (CD8+ T-cells, natural killer cells) with the tumour epithelial compartment, which may result from a barrier formed from a particular composition of tumour ECM (shown as the orange and green fibres in the illustration above). The ECM barrier reduces immunotherapy response.

Over the last decade, an increasing body of evidence points towards the TME as a major predictor of immunotherapy success. From tumour tissue analyses, several immune phenotypes have been defined that are predictive of response to immunotherapy (6). Evidence of high levels of intra-tumour immunological activity such as T cell infiltrates before treatment correlates with clinical benefits of ICB therapy, suggesting that a pre-existing anti-tumour immune response has been hijacked by the tumour cells but can be reinvigorated by immunotherapies (3, 7). Accordingly, solid tumours can be classified as ‘inflamed’ when demonstrating abundant tumour infiltration with immune cells and numerous inflammatory mediators. Or, on the contrary, they can be classed as ‘immune-desert’, where there is little to no immune infiltration into the tumour (8). An additional classification is the ‘inflamed-excluded’ phenotype, which distinguishes tumours where immune cells are retained in the surrounding stroma, reflecting a blockage in tumour penetration. Therefore, T-cell migration through the tumour stroma appears to be the rate-limiting step, as the majority of responding tumours across cancers have an inflamed phenotype, with the inflamed-excluded phenotype making up the majority of non-responders (**Figure 1**).

The classification of the TME immune phenotype is associated with specific underlying biological mechanisms (8). In particular, tumours with an ‘inflamed-excluded’ phenotype are associated with increased matrisome deposition (**Figure 1**). The matrisome is the collective of the extracellular matrix (ECM), the secretome and associated proteins (9). Recently, accumulated data from tumour biopsies has highlighted cancer-induced matrisome remodelling in immune evasion and immunotherapy failure. Salmon, Donnadieu and colleagues established that matrisome architecture impeded the migration of T cells in the stroma limiting their access to tumour (10). Using live cell imaging in human lung cancer, they revealed that aligned fibres of matrix surrounding the tumours dictated the motile behaviour of T cells and their capacity to infiltrate. Further real-time imaging analyses showed the matrisome could trap CD8+ T cells within the tumour stroma of lung, ovarian and pancreatic carcinomas (11, 12). Similarly in breast tumours, Acerbi et al. established that biomechanical changes underlying matrisome remodelling were induced by the abundant number of infiltrating immune cells at the invasive front of the tumour (13).

Meanwhile, Chakravarthy et al., using pan-cancer analyses, reported a gene signature to predict failure of response to ICB, which related to matrisome dysregulation (14). They suggested that matrisome remodelling is an immune evasion mechanism mediated by TGF-β-activated fibroblasts to a subtype of cancer-associated fibroblast (CAF). This paradigm is consistent with a recent recurrent observation of a CAF subtype, which may promote an immunosuppressive environment through depositing a high amount of matrisome (15, 16). In addition, more transcriptional analyses identified a similar matrisome remodelling signature in resistance to cancer immunotherapies and failed immune responses, suggesting its use in diagnosis and precision therapies (17). In melanoma, quantification of the expression of proteins within the stromal matrisome before treatment was associated with poor response to ICB and poor prognosis, confirming the potential of targeting the matrisome (18).

In our own work, we identified from multilevel analysis a matrisome signature common across thirteen solid cancers (19). This signature defined a tissue matrisome composition which associated with poor prognosis, immunosuppressive cell phenotypes and negatively correlated with cytotoxic T cell signatures. At the same time, two back-to-back studies found that inhibition of matrisome deposition by targeting TGF-β could limit immune evasion and sensitise tumours to anti-PD-L1 immunotherapy in pre-clinical models (20, 21). Together, these studies suggest that a specific composition of tumour matrisome may form a barrier to anti-tumour immunity. This immune-barrier may be physical, through alignment of fibrous proteins and stiffening of the tissue, or also through receptor-ligand interactions. By identifying components of the matrisome contributing to the immune barrier, it may be possible to identify targetable molecules. The inhibition of these molecules will lead to an altered matrisome composition, permitting T cell contact with malignant cells, therefore improving cytotoxic tumour immunity. Such an approach may convert an inflamed-excluded phenotype to an inflamed phenotype and result in better response to immunotherapy.

From our matrisome signature (19) and the signature identified by Chakravarthy et al (14), versican within the matrisome correlated strongly with immune suppression and immunotherapy failure within the TME. Versican is an extracellular matrix proteoglycan that interacts with other ECM components and cells to influence disease phenotype. From transcriptomic studies versican expression increases in common solid cancers including ovarian, pancreatic, breast, lung, esophageal, and colorectal (14, 19, 22–24). While other matrisome molecules associate with disease progression and immune suppression such as several collagens, fibronectin, and matrix proteases, versican stands out because of its impact on several different cellular events that form the basis for the progression of cancer, including proliferation, metastasis, invasion, and immunity (25–27). Here we focus on versican, because it has been shown to have direct association with immune cell phenotype and trafficking in inflammatory diseases and development, and we consider here how these roles for versican may translate to cancer immunity. Overall our review suggests versican could be a component within tumours displaying an inflamed-excluded phenotype, highlighting its potential as a target for cancer immunotherapy.

## Versican in the ECM

### The Structure of Versican

**Figure 2:**
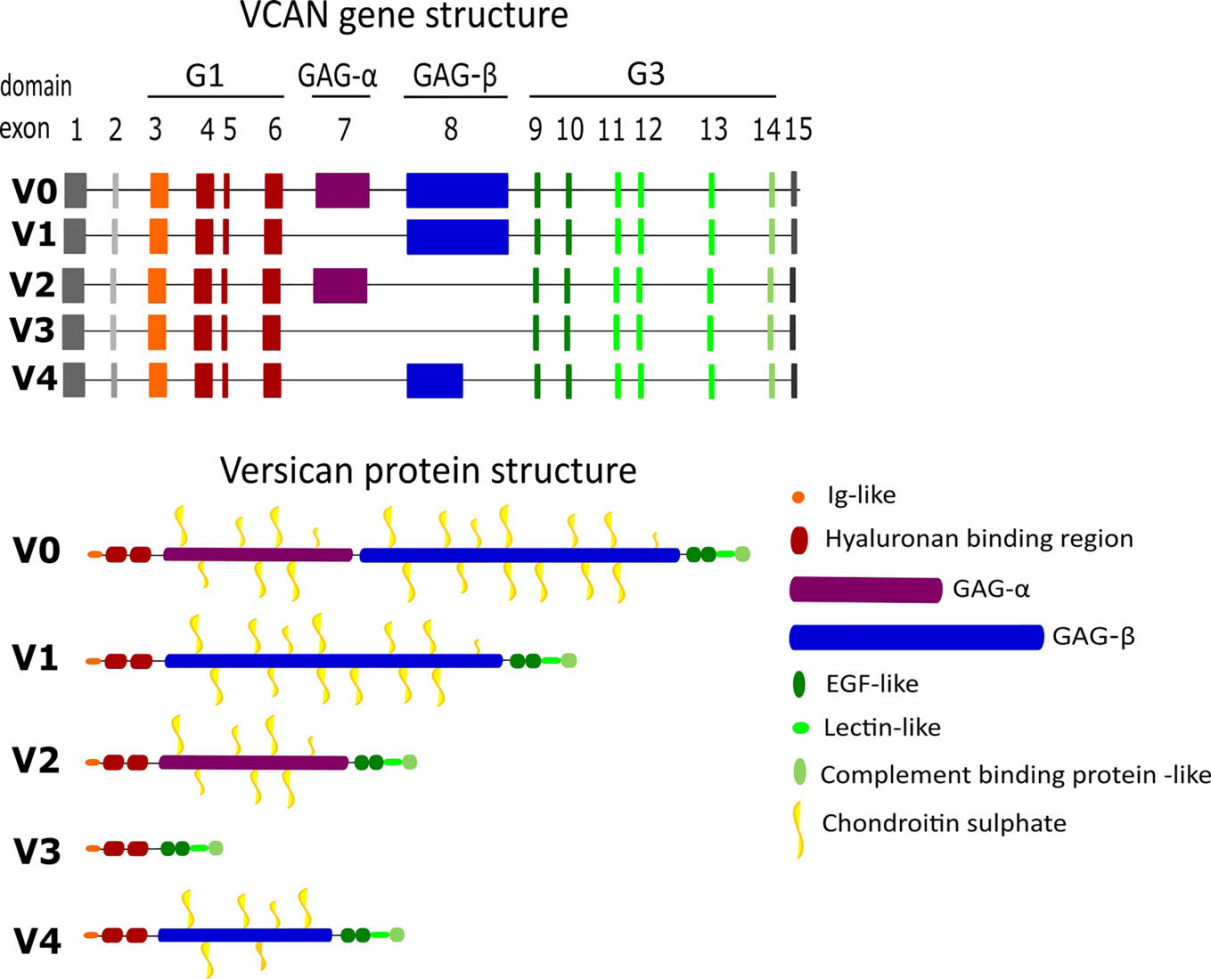
Gene and protein structure of the different versican isoforms. Versican is formed of multiple motifs and domains which contribute to its binding abilities.

Versican is a member of the proteoglycan family of matrisome proteins. These are structurally distinct proteins with post-translational modifications (PTM) of polysaccharides containing amino sugars, known as glycosaminoglycans (GAGs) (28). Versican has three distinct regions comprising the core protein (29). The N-terminal G1 domain contains a hyaluronan-binding region which binds to hyaluronan to form the ‘hyalectin’ complex, and the C-terminal G3 domain consists of two epidermal growth factor-like repeats, a calcium-dependent C-type lectin binding domain and a complement binding protein-like motif (**Figure 2**). Between these domains, the core protein consists of two GAG binding regions; the α-GAG and β-GAG domains, from which the GAG chondroitin sulphate (CS) extends following PTM during golgi processing (30). Versican interacts with a diverse array of ECM ligands and cell-surface molecules, thus contributing significantly to the versatile function of the molecule (31, 32).

Sequencing of the structure of versican has revealed five splice variants (**Figure 2**), leading to a core protein size ranging from approximately 74-370 kDa (31, 33). The isoforms are generated from alternative splicing of exons 7-8, which encode the α-GAG and β-GAG domains respectively. The largest isoform is V0 with both GAG domains intact, and 17-23 potential CS modification sites. V1 and V2 are smaller isoforms, lacking the α-GAG and β-GAG regions respectively, and therefore present fewer available CS modification sites, from 12-15 in V1 and 5-8 in V2. The smallest isoform V3 lacks both GAG domains, and thus has no CS binding sites to be functionally defined as a proteoglycan (34, 35). Awareness of this structural variation has helped to elucidate domain-specific functions; most notably the absence of CS-modification sites in V3 has enabled clarification of the roles of these regions (36). An analysis of isoforms by Kischel et al. in 2009, revealed the fifth structural variant V4, comprising G1, G3 and a truncated β-GAG domain. RT-PCR cloning analyses revealed V4 in human breast cancer lesions, but it was barely detectable in normal breast tissue (37).

The isoforms are structurally distinct at the translational and post-translational levels (38), leading to differential roles in normal homeostasis, inflammation and malignancy. During embryogenesis, isoforms V0 and V1 are found within the developing heart and brain. Within adult tissues, these isoforms are predominant in inflammatory environments such as tumours and provide pro-proliferative and anti-apoptotic functions (25). V2 expression is prominent in the brain, where it is the major isoform following embryonic development (39–43). Unlike V0 and V1, V3 does not appear to be elevated in disease but likely counteracts the effects of V0/V1 on cell phenotype, acting as a dominant negative isoform (25, 26). Concerning V3, several studies show its mRNA expression in a variety of tissues, but only a few have identified deposits of the protein due to the lack of a V3-specific antibody. Its biological roles have been determined mainly through transgenic overexpression models. These models indicate that V3 expression increases elastic fibre deposition, reduces hyaluronan accumulation and promotes an anti-inflammatory phenotype within vascular tissues (44, 45). Versican splice variants differ greatly in length and in interaction with their linking partners, predicating distinct impacts on matrix modelling. In particular, these observations highlight the importance of the CS binding domains in determining the functional roles of the isoforms.

### Versican and the TME Matrisome

The binding capabilities of versican within normal physiology and non-malignant tissues further complicate the potential roles it can play within the TME. Versican interacts with multiple proteins and carbohydrates within the ECM through different domains as shown in **Figure 2**. The predominant binding partner for versican is the GAG hyaluronan. Within most tissues, versican is bound to hyaluronan with high levels of co-localisation identified in malignant tissues. Hyaluronan production is upregulated in tumours and associated with tumour progression (46). Together the versican-hyaluronan aggregates form cable-like lattices, increasing the viscosity of tissues and decreasing matrix permeability (32, 47). Structural variation between versican isoforms contributes to changes in the size of versican-hyaluronan aggregates, influencing tissue volume (48) and macromolecular organisation (49, 50). The aggregate can bind to CD44 to form a supramolecular complex, reducing the presence of elastic fibres (33). These complexes can be stabilised by the presence of link proteins such as HAPLN1. Studies have shown that HAPLN1 can affect immune infiltration as well as tumour dissemination in aged TMEs (51, 52).

In addition to hyaluronan, versican also interacts with tenascin-R, type I collagen, fibulin-1 and -2, fibrillin-1, fibronectin, and P- and L-selectins (32, 53). Through its lectin-binding domain, versican connects fibrillin microfibrils to hyaluronan-rich matrices, providing elasticity, which is essential within vascular tissues. The interaction of versican with fibronectin occurs through the G3 domain (32). With both fibronectin and versican being overexpressed in tumours this interaction may be critical for the formation of a pro-tumour matrix.

The inability of versican to interact with proteins in the ECM has been linked to specific disorders. Germline mutations within the versican-binding domain of FBN1 can result in severe forms of Marfan syndrome; an inherited connective tissue disorder, possibly indicating the loss of a stable interaction between microfibrils and versican as a pivotal contributor to this fibrillinopathy (32, 53). Similarly, mutations in the versican gene are solely responsible for the rare Wagner Syndrome disease characterised by a progressive break down of the retina, highlighting the essential role versican plays in matrix stability and scaffolding.

The following sections will outline the role of versican within immune cell trafficking and how its expression within the TME may be promoting an inflamed-excluded phenotype which associates with a poor response to immunotherapy.

### Versican and Immune Trafficking

**Figure 3:**
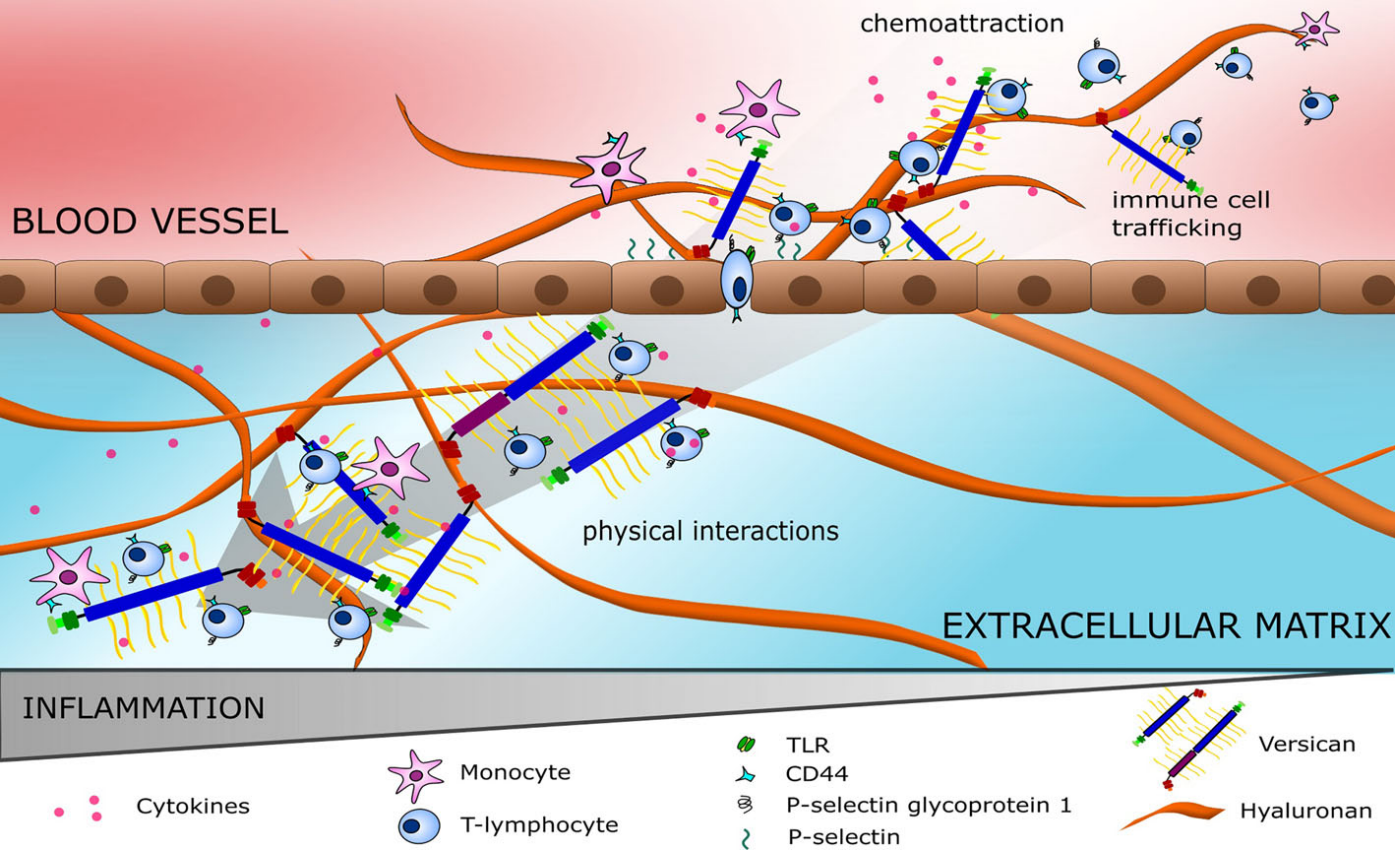
Immune cell trafficking on highways of versican. Versican (V0/V1) attracts pro-inflammatory cytokines forming a gradient to guide immune cells. Immune cells bind to versican and hyaluronan *via* receptors such as TLRs, CD44 and P-selectin glycoprotein 1, mediating cell rolling followed by extravasation into the ECM. Within the ECM, a cytokine gradient is also observed with immune cells locating to areas with high versican expression.

Altered immune cell trafficking and polarisation of immune cell phenotype is a prominent feature of most cancers and several studies have identified a role for versican in these processes (24, 27, 54). During an inflammatory response, versican interacts with several immune cell membrane proteins including CD44, integrin-β1, and P-selectin glycoprotein ligand 1, to guide leukocyte trafficking; as illustrated in **Figure 3** (32, 55). Versican interacts with CD44 indirectly through hyaluronan and directly *via* its CS chains (26), these interactions are dependent on the sulphation of the CS chains (32, 56, 57). These interactions mediate rolling of T cells on endothelial cells during homing (58). In addition, versican is also important in the process of leukocyte adhesion during trafficking (59). T cells avidly adhere to the versican- and hyaluronan-rich matrisome that is produced during inflammation. This adhesion can inhibit T cell spreading and migration (59). As well as T cells, monocytes have also been shown to adhere to versican, slowing their migration through the inflamed tissue. The addition of monoclonal antibodies to block the G1 domain was found to inhibit this adherence, suggesting that the interaction of versican with hyaluronan is important for immune cell adhesion (60). The G3 domain also plays a role in immune cell trafficking with P-selectin glycoprotein ligand 1 binding to the G3 domain to induce leukocyte aggregation, this is important for immune activation and cell signalling (61).

The negatively-charged GAGs of versican attract various positively-charged molecules, including growth factors (e.g., VEGF, TGF-β), chemokines (e.g., CXCL1, -2, -3) and cytokines (e.g., IL-1, IL-2, IL-4) (62), producing reservoirs of immune signals that result in gradients which direct the inflammatory response and immune cell phenotype within a tissue (32). For example, Masuda *et al.* found CCL2-chemotactic gradients are established through binding to versican CS glycosylation sites (63). CCL2 binds to CCR2 and guides the movement and recruitment of monocytes and macrophages across the TME (64). Once recruited, versican-mediated monocyte activation occurs *via* CD44 receptors and integrins. Additionally, polarisation towards a more tumour-promoting Th2 and pro-fibrotic macrophage phenotype can occur where CCL2 is present (32, 63). Chemokine binding to CS chains is selective, with attachment only occurring on chains with the ability to bind to L-selectin (56). Through chemokine gradients and direct interaction, versican could be considered a major component in the roads and highways that immune cells use to traverse a tissue. In cancer, these versican highways travelled by immune cells may actually serve to protect tumour cells from the immune response. The hyaluronan- and versican-rich pericellular matrix often expressed around malignant cells may be a shield from patrolling natural killer cells and other lymphocytes, as well as preventing antibody-dependent cell-mediated cytotoxicity (65, 66).

### Versican Influences Exclusion

Versican expression may promote tissue stiffness in tumours and other diseases, such as sclerosis, that result in altered tissue composition (67, 68). The mechanism likely involves the versatile binding properties of versican to link other ECM proteins together which enables the formation of fibrous structures and increases in tissue stiffness. For example, versican can bind to collagen which maintains fibre density that enables tissue rigidity (51, 69). In our own work, we found versican was a member of a signature of twenty-two matrisome molecules that associated with tumour tissue stiffness at both the gene and protein levels (19). Interestingly, this signature also associated with T cell inhibition, and fibrotic ‘barriers’ have been suggested to physically impede T cell infiltration *in vivo*, as may be the case in pancreatic cancer (70). Immunotherapeutic response is correspondingly poor in stiff tumour tissue and approaches to enhance tumour-infiltrating lymphocyte entry are highly sought after. It may also be that versican influences the poor penetrance of desmoplastic stroma that prevents chemo- and immune-therapeutic agents from reaching the tumour itself, however it remains unclear if this is the result of the physical stiffness of the tissue forming a barrier, or *via* receptor ligand interactions between molecular patterns on the mesh of matrisome proteins and complementary receptors on immune cell infiltrate, or a combination of the two.

### Versican Alters Immune Phenotype

As well as providing a ‘shield’, malignant cell-expressed versican could directly alter immune cell phenotype though engagement of cell receptors. This has been documented for both innate and adaptive immune cells. In the former, versican expression is associated with more tumour-associated macrophages (TAMs) which tended to have a pro-tumour phenotype (71), which could be reversed by silencing versican expression. This interaction of versican with macrophages may be isoform-specific, as found in a study with ovarian cancer cell lines where silencing of the V1 isoform reduced the activation of TLR2, -6 and CD14 on macrophages (72). Versican stimulation of TLR2 on macrophages increases the expression of TNF-α, which can sometimes promote tumour progression (73, 74) through mechanisms such as PD-L1 upregulation on both myeloid and tumour cells (75, 76). TLR2 activation by versican also reduces dendritic cell anti-tumour response through IL-10 and IL-6 signalling, leading to the mitigation of conventional dendritic cell responses required for further downstream T cell activation (77, 78).

**Figure 4:**
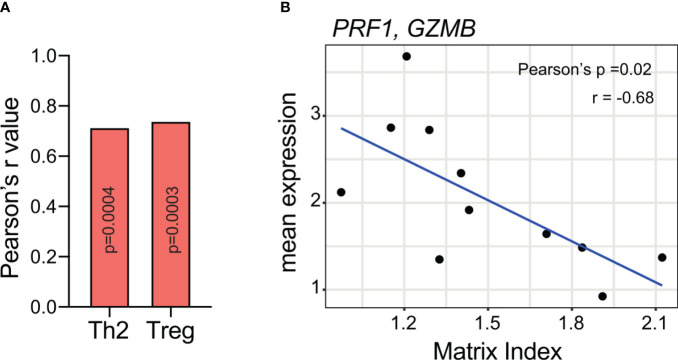
A matrisome signature containing versican **(A)** positively correlates with Th2 and T regulatory expression and **(B)** negatively correlates with cytotoxic T cell markers. Meta-analysis on RNA-seq data from Pearce et al. (19). Analysis was completed on 33 patient samples of high-grade serous ovarian cancer omental metastasis. The matrix index refers to a signature of 22 matrisome genes identified in the paper with values determined from a ratio of upregulated and downregulated genes. Versican is one of six genes that were upregulated in tumours. Spearman rank correlation was used to compare matrix index values from immune cell expression (19, 79). P-values were FDR corrected using the Benjamini & Hochberg method. The average expression of PRF1 and GZMB (genes associated with cytotoxic T cells) was correlated to matrix index values from 11 poor prognostic patients using Pearson’s correlation. Th2, T helper cells; Treg, T regulatory cells; PRF1, perforin 1; GZMB, granzyme B.

In the adaptive immune response, versican can polarize the CD4+ T cell response to a T regulatory phenotype. One study found that in versican-deficient tumours, pleural T regulatory cell numbers were reduced along with tumour mass in a preclinical model of mesothelioma (71). These findings are supported by our own work where we identified a specific composition of tumour matrisome, of which versican is a component, that positively and significantly correlated with T regulatory and Th2 cell signatures, and negatively correlated with cytotoxic cell signatures (**Figure 4**) (19).

Whilst versican stimulates gradients of cytokines in tissues, its expression is also regulated by cytokines including TGF-β, PDGF, IL-1α and IL-1β (47, 55, 60, 80, 81). In addition, versican itself has the ability to affect inflammatory cytokine release in stromal cells and immune cells, therefore appropriate cytokine-mediated versican production may promote the next inflammatory response (55). In this case versican acts as a TLR2 agonist; stimulating TLR2 and its co-receptors TLR6 and CD14 to release pro-inflammatory molecules such as TNFα and IL-6, inducing macrophage activation (81–85). This positive feedback loop leads to prolongation of an inflammatory environment.

### Cellular Origin of Versican Impacts Function

Elevated levels of versican in the tumour are mostly expressed by either malignant cells and/or tumour-associated stromal cells (86, 87). In a recent study on the cellular origins of matrisome proteins in pancreatic cancer, levels of versican produced by malignant cells were associated with poor patient survival, while in an investigation in node-negative breast cancer, relapse correlated with versican stromal levels deposited by mammary fibroblasts (88). The secretion of TGF-β1 by malignant cells led to an upregulation of versican secretion by mammary fibroblasts (89). Versican is expressed by myeloid cells stimulated by hypoxic (90) and inflammatory cytokines (91, 92). These studies suggest that the cellular origin of versican may differ across cancer types. The cellular origin may be important because it could account for the variation found in translational and post-translational structures of versican, that in turn dictates its function. Inhibiting versican synthesis in specific cells may be an interesting way to investigate this hypothesis.

### Isoforms of Versican Have Diverse Roles

The unique structures of the different isoforms of versican impact their binding abilities and strengths. This can lead to variations in the roles of the isoforms within the tissue. In cancer tissues, versican appears to be expressed mostly in the V0 and V1 isoforms (88, 93, 94), and silencing of these isoforms has a direct effect on cancer progression, migration and invasion (95, 96). The increase in migration seen when versican expression is inhibited may result from an increase in cell adhesion to matrisome proteins collagen I and fibronectin (95). The V2 isoform appears to stimulate vascularisation within tumours, which may reduce tumour proliferation in the short term (the opposite of what has been recorded for V0 and V1 isoforms), whilst supporting cancer cell survival (97). The few studies documenting V3 upregulation in malignancy show the isoform demonstrating contrasting roles based on tissue context. For example, V3-overexpressing melanoma cells exhibited reduced growth potential *in vivo*, however, hyaluronan-CD44-dependent migration was enhanced (98). Deeper analysis revealed a dual role for this isoform, with enhanced lung metastasis suggesting a pro-metastatic function despite an inhibition of primary tumour growth (99). V4-specific properties are less extensively mapped; nevertheless, it is thought that elevated V4 in the TME may contribute to tumour progression through TGF-β1 derived from primary breast fibroblasts (37).

### Versican Proteolysis Reverses Exclusion

In addition to the isoforms of versican, products from its proteolytic cleavage within the TME termed versikines are implicated in generating anti-tumour immunity. Recently, studies have focused on the functional characterisation of versikine, a Glu441-Ala442-cleaved V1 N-terminal fragment. This fragment is produced by MMP-mediated V1 degradation, principally involving ADAMTS1, -4 and -5 (100–103). Proteolysis of non-V1 isoforms can also form N-terminal fragments (101) but they have not been as extensively researched. Versikine can engage G1 cell-surface targets such as TLR2 on macrophages stimulating anti-tumour immunity, emphasising its role as a damage-associated molecular pattern (DAMP) (104, 105).

**Figure 5:**
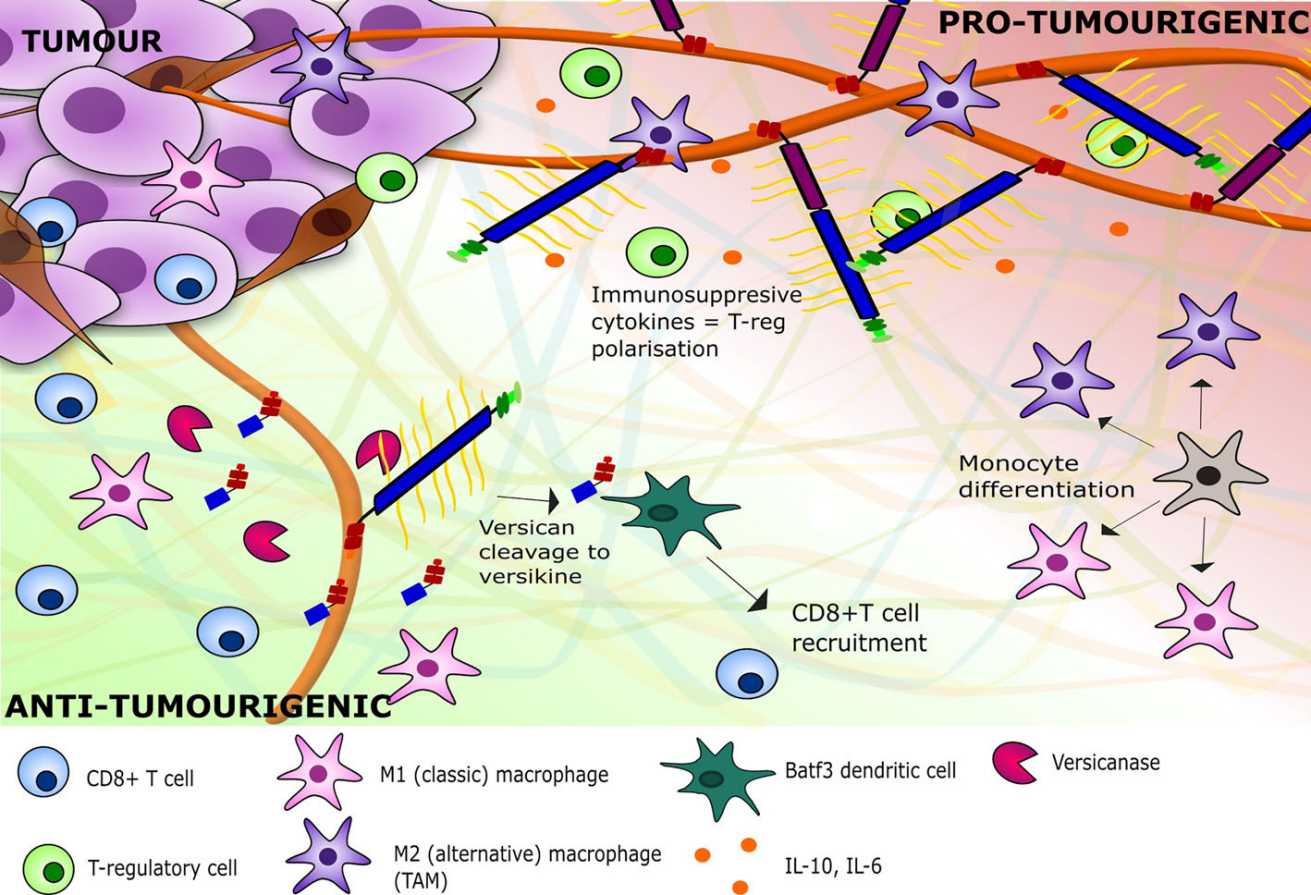
A depiction of the inflammatory milieu of versican V0/V1 and versikine and their respective effects on tumour immunity. V0/V1 presence leads to the stimulation of immune-suppressive cytokines within the TME, leading to a pro-tumourigenic environment with polarisation of immune cells to TAMs and regulatory T cells. The breakdown of V1 by versicanases such as ADAMTS1 leads to an adaptive immunity against the tumour with recruitment of CD8+ T cells and polarisation of monocytes towards M1 macrophages. ADAMTS1, ADAM Metallopeptidase with Thrombospondin Type 1 Motif 1; TAM, Tumour-associated macrophage.

Proteolysis of versican has been associated with CD8+ T cell migration in inflammatory diseases and cancer (104–108). Notably in myelomas, intense proteolysis of macrophage-derived V1 following stromal ADAMTS1 secretion has been associated with CD8+ T cell tumour infiltration (104). Purified versikine induces IL-6 and IL-1β expression in myeloma marrow-derived macrophages through partially TLR2-independent mechanisms *in vitro (* 105). Furthermore, versikine may also promote tumour cell apoptosis (105, 109). Through these mechanisms, versikine can promote CD8+ T cell inflammation and activation, which blocks the tolerogenic polarisation directed by intact versican (105). The proteolytic cleavage of versican can therefore induce a general immunogenic response within the TME as summarised in **Figure 5**, which may be helpful in potentiating recently emerging T cell activating immunotherapies. In addition, versican proteolysis may serve as a positive prognostic biomarker, as shown in colorectal cancer, where versikine expression was associated with a high CD8+ T cell infiltrate, the absence of early metastatic invasion, and prolonged survival (105).

### Targeting Versican

Targeting the tumour matrisome is already underway in preclinical and early clinical studies (110, 111). One lesson we have learnt from these studies is that the matrisome has a dual function in disease progression, acting as both a barrier to tumour growth, but also as a barrier to anti-tumour immunity (112). Therefore, targeting major constituents of the tumour matrisome can inadvertently help support tumour growth presumably by removing a barrier to tumour cell migration. These results indicate that we must be more selective in our approach to matrisome targeting to limit deleterious and unwanted effects and use combinatorial treatment approaches that are ready to attack tumour once the matrisome barrier comes down. With this in mind, there may be therapeutic potential in targeting versican whose roles in immune exclusion we have shown here include providing vectors for immune cell trafficking through biochemical cues or contributing towards a stiff tumour matrisome through biophysical cues. Important in our consideration of how we target versican is the diverse roles its isoforms and fragments may play in disease progression. Further, it may be that many properties of versican in immune exclusion are mediated through the post-translational modifications of CS across the protein backbone of the α- and β-GAG regions. These CS modifications may prove to be attractive targets because they can be removed *in situ* using enzymes – an approach that is currently showing promise for other types of tumour glycosylations (113). From the data acquired by multiple groups studying versican in various tumours, it is evident that the isoforms V0 and V1 are the most dominant and have the greatest impact on tumourigenesis. The commonality between these isoforms is the large length and presence of the β-GAG domain. To target these isoforms there are multiple sites on which to focus. The first site is the hyaluronan binding site. As shown in Evanko *et al.*, the binding of versican to hyaluronan causes clumping of immune cells, whilst the use of an antibody to inhibit this interaction restored cell migration (59). Therefore, targeting this interaction *via* blocking or degrading the binding sites can be seen as a potential treatment option. The second potential target site would be the CS chains. The CS chains support gradient formation and cytokine localisation, and therefore targeting the synthesis of the chains or enzymatic breakdown of the chains may impact chemotaxis of immune cells across the matrisome. Chondroitin-6-sulphate (C-6-S) has been identified to be synthesised at a greater proportion in tumour tissues compared to chondroitin-4-sulphate or its unsulphated counterpart. The conformation of C-6-S is thought to make it more accessible for binding, making it more favourable within the matrisome. In addition, C-6-S was found to inhibit IL-6 secretion by macrophages and skewed them towards an M2-like phenotype (114, 115). The third target site is the GAG binding domains. As mentioned previously, the β-GAG domain has a greater association with tumour progression and immune cell localisation. Targeting the protein through the GAG binding domain can also lead to the formation of versikine structures which could potentially improve immune cell infiltration in comparison to the full form of the protein.

## Summary

The tumour matrisome plays a role in limiting the success of immunotherapy. The formation of an immune-excluded tumour inhibits cytotoxic cell contact with malignant epithelial cells. Versican expression is associated with a composition of matrisome that associates with poor prognosis and failure of immunotherapy response. We therefore propose that versican is a potential candidate for targeting to improve the immune infiltration and immunotherapy response, which we have explored in this review.

Versican does have contrary roles within the TME as it is involved in both tumour progression and inhibition. These opposing functions can be explained by the diverse structures of the versican protein, which are expressed in a variety of isoforms, post-translational modifications, and fragmentation products, and may be dependent on the cell of origin and the inflammatory environment present within a tumour. The functional roles for versican in cancer are broad, including impacting proliferation, survival, invasion, metastasis, and inflammation. We have focused here on how versican may contribute to immune-excluded tumours that have poor response to therapy and tumour-supporting innate and adaptive immune cell phenotypes, and identified two mechanisms though which this occurs, by biochemical and biophysical cues.

In summary, targeting versican, and in particular targeting specific structural forms of versican can maximise the therapeutic potential whilst minimizing deleterious effects that can be associated with tumour matrisome targeting. We conclude here that targeting the V0/V1 isoforms likely *via* the β-GAG domain, and specifically the modifications of CS may provide the most specific targeting. However, it is important to note that as further studies on versican isoforms become available, other isoforms may prove to be more desirable targets, for example the V4 isoform which appears to be highly disease-specific from the limited studies currently available. Whilst our focus here has been on versican as a specific target within the matrisome, other proteins may also prove to be important components within the immune barrier. For example, both collagen and fibronectin have been highlighted to form tight networks that can limit space for cell movement (10). Therefore, identifying the specific components of the barrier, such as versican, and understanding its structural variations in tumours can aid in developing novel approaches that may improve immunotherapy response for many people across many cancer types.

## Author Contributions

PH wrote and revised the manuscript. VG wrote and revised the manuscript. CA wrote the manuscript. TW edited and revised the manuscript. OP planned, wrote, edited and revised the manuscript. All authors contributed to the article and approved the submitted version.

## Funding

OP is a recipient of a Cancer Research UK fellowship (grant number A27947). PH and OP are recipients of a Barts Charity and Against Breast Cancer PhD studentship (grant number MGU0499). TW was funded by National Institutes of Health grants R01 AI 130280, R01 DK096087, and U19 AI125378. Work in the Wight laboratory was also supported by the Ann Ramsey-Jenkins and William M. Jenkins Fellowship for Matrix Biology.

## Conflict of Interest

The authors declare that the research was conducted in the absence of any commercial or financial relationships that could be construed as a potential conflict of interest.

## Publisher’s Note

All claims expressed in this article are solely those of the authors and do not necessarily represent those of their affiliated organizations, or those of the publisher, the editors and the reviewers. Any product that may be evaluated in this article, or claim that may be made by its manufacturer, is not guaranteed or endorsed by the publisher.

## Abbreviations

CAF, cancer-associated fibroblast; CS, chondroitin sulphate; ECM, extracellular matrix; GAG, glycosaminoglycan; ICB, immune checkpoint blockade; PD-L1, Programmed death receptor-1 ligand; PTM, post-translational modifications; TAM, tumour-associated macrophages; TME, tumour microenvironment.
